# Expert opinion paper on cardiac imaging after ischemic stroke

**DOI:** 10.1007/s00392-021-01834-x

**Published:** 2021-06-18

**Authors:** Renate B. Schnabel, Stephan Camen, Fabian Knebel, Andreas Hagendorff, Udo Bavendiek, Michael Böhm, Wolfram Doehner, Matthias Endres, Klaus Gröschel, Andreas Goette, Hagen B. Huttner, Christoph Jensen, Paulus Kirchhof, Grigorios Korosoglou, Ulrich Laufs, Jan Liman, Caroline Morbach, Darius Günther Nabavi, Tobias Neumann-Haefelin, Waltraud Pfeilschifter, Sven Poli, Timolaos Rizos, Andreas Rolf, Joachim Röther, Wolf Rüdiger Schäbitz, Thorsten Steiner, Götz Thomalla, Rolf Wachter, Karl Georg Haeusler

**Affiliations:** 1 Department of Cardiology, University Heart and Vascular Center Hamburg, Hamburg, Germany; 2 German Centre for Cardiovascular Research (DZHK), Partner site Hamburg/Kiel/Lübeck, Hamburg, Germany; 3 Atrial Fibrillation NETwork (AFNET) e.V., Münster, Germany; 4 Department of Cardiology and Angiology, University of Berlin, Charité-Universitätsmedizin Berlin, Berlin, Germany; 5 Klinik und Poliklinik für Kardiologie, Universitätsklinikum Leipzig , Leipzig, Germany; 6 Department of Cardiology and Angiology, Hannover Medical School, Hannover, Germany; 7 Internal Medicine III, Cardiology, Angiology and Intensive Care Medicine, University Hospital of Saarland, Saarland University, Homburg (Saar) , Germany; 8 Berlin Institute of Health, Center for Regenerative Therapies, and Department of Cardiology (Virchow Klinikum), Charité-Universitätsmedizin Berlin, Berlin, Germany; 9 German Centre for Cardiovascular Research (DZHK), Partner Site Berlin, Berlin, Germany; 10 Center for Stroke Research Berlin (CSB), Charité Universitätsmedizin Berlin, Berlin, Germany; 11 Klinik Und Hochschulambulanz Für Neurologie Mit Abteilung Für Experimentelle Neurologie, Charité-Universitätsmedizin Berlin, Berlin, Germany; 12 German Center for Neurodegenerative Diseases (DZNE), Partner Site Berlin, Berlin, Germany; 13 ExcellenceCluster NeuroCure, Berlin, Germany; 14 Department of Neurology, University Medical Center of the Johannes Gutenberg University Mainz, Mainz, Germany; 15 Department of Cardiology & Intensive Care Medicine, St. Vincenz Hospital Paderborn, Paderborn, Germany; 16 Department of Neurology, University Hospital Gießen, Gießen, Germany; 17 B. Braun Ambulantes Herzzentrum Kassel MVZ GmbH, Kassel, Germany; 18 Ruhr University Bochum, Bochum, Germany; 19 Institute of Cardiovascular Sciences, College of Medical and Dental Sciences, Medical School, University of Birmingham, Edgbaston, Birmingham, UK; 20 Department of Cardiology and Vascular Medicine, GRN Hospital Weinheim, Weinheim, Germany; 21 Department of Neurology, University Medical Center Goettingen, Goettingen, Germany; 22 Comprehensive Heart Failure Center and Department for Medicine I, University Hospital Würzburg, Würzburg, Germany; 23 Department of Neurology, Vivantes Klinikum Neukölln, Berlin, Germany; 24 Department of Neurology, Klinikum Fulda, Universitätsmedizin Marburg – Campus Fulda, Fulda, Germany; 25 Department of Neurology, Goethe-University Hospital Frankfurt, Frankfurt, Germany; 26 Department of Neurology & Stroke, Eberhard-Karls University Tübingen, Tübingen, Germany; 27 Department of Neurology, Heidelberg University, Heidelberg, Germany; 28 Department of Cardiology, Kerckhoff-Heart-Center, Bad Nauheim, Germany and Campus Kerckhoff Justus-Liebig-University, Gießen, Germany; 29 Department of Neurology, Asklepios Klinik Hamburg Altona, Hamburg, Germany; 30 Department of Neurology, Evangelisches Klinikum Bethel, Universitätsklinikum OWL der Universität Bielefeld, Campus Bielefeld-Bethel, Bielefeld, Germany; 31 Department of Neurology, Klinikum Frankfurt Höchst, Frankfurt, Germany; 32 Department of Neurology, University Medical Center Hamburg-Eppendorf, Hamburg, Germany; 33 University Medical Center Goettingen, Göttingen, Germany; 34 Department of Neurology, University Hospital Würzburg, Josef-Schneider-Str. 11, 97080 Würzburg, Germany; 35 Department of Neurology and Clinical Neurophysiology, Klinikum Lüneburg, Lüneburg, Germany; 36 Hertie Institute for Clinical Brain Research, Eberhard-Karls University Tübingen, Tübingen, Germany

**Keywords:** Cardiac imaging, Echocardiography, Ischemic Stroke, Transient ischemic attack, Expert opinion, Magnetic resonance imaging, Computed tomography

## Abstract

This expert opinion paper on cardiac imaging after acute ischemic stroke or transient ischemic attack (TIA) includes a statement of the “Heart and Brain” consortium of the German Cardiac Society and the German Stroke Society. The Stroke Unit-Commission of the German Stroke Society and the German Atrial Fibrillation NETwork (AFNET) endorsed this paper. Cardiac imaging is a key component of etiological work-up after stroke. Enhanced echocardiographic tools, constantly improving cardiac computer tomography (CT) as well as cardiac magnetic resonance imaging (MRI) offer comprehensive non- or less-invasive cardiac evaluation at the expense of increased costs and/or radiation exposure. Certain imaging findings usually lead to a change in medical secondary stroke prevention or may influence medical treatment. However, there is no proof from a randomized controlled trial (RCT) that the choice of the imaging method influences the prognosis of stroke patients. Summarizing present knowledge, the German Heart and Brain consortium proposes an interdisciplinary, staged standard diagnostic scheme for the detection of risk factors of cardio-embolic stroke. This expert opinion paper aims to give practical advice to physicians who are involved in stroke care. In line with the nature of an expert opinion paper, labeling of classes of recommendations is not provided, since many statements are based on expert opinion, reported case series, and clinical experience.

**Supplementary Information:**

The online version contains supplementary material available at 10.1007/s00392-021-01834-x.

## Introduction

Whereas imaging of the brain and the brain-supplying arteries as well as electrocardiogram (ECG) monitoring is standard in stroke diagnostics [1, 2], discussion of the clinical impact and appropriate method of cardiac imaging is ongoing [3, 4]. As the available evidence is limited, present guideline recommendations on cardiac imaging after ischemic stroke or TIA remain vague and in principle, the indication of cardiac imaging remained at the discretion of the treating physician. As an example, the European Stroke Organization (ESO) guideline issued in 2008 recommends echocardiography in selected stroke patients, e.g. in case of suspected cardioembolism (Class III, Level B recommendation) [5]. However, no recommendations were given as to the choice of transthoracic (TTE) or transoesophageal (TOE) echocardiography. In a recent consensus statement from the ESO-Karolinska Stroke Update Conference, TTE was considered the primary choice for cardiac imaging (Grade A), while TOE and bubble test-transcranial Doppler were recommended in patients with an embolic stroke of undetermined source (ESUS) for PFO detection (Grade A) as well as TOE over TTE to detect aortic atheroma (Grade C) [6]. Recently updated guidelines on acute stroke management consider echocardiography reasonable in selected ischemic stroke patients to guide secondary stroke prevention (American Heart Association, Class IIa recommendation, expert opinion) and to determine whether eligibility criteria for PFO closure are met (American Heart Association, Class IIa recommendation) or in case of cryptogenic stroke (German Society of Neurology & German Cardiac Society, Class Ia recommendation) [1, 7]. The American Society of Echocardiography guidelines were published in 2016 and recommend routine use of TTE as a screening tool for potential cardiac sources of embolism, while TOE might be considered as an initial or supplemental test in specific cases, e.g. suspicion for endocarditis [8]. Cardiac CT and MRI should be reserved to selected patients with high suspicion for cardioembolism and inconclusive results after echocardiography. Due to its semi-invasive nature, TOE is not recommended if potential results will not change therapeutic decisions [8]. The Canadian Stroke Best Practice Recommendations for Acute Stroke Management, issued in 2018, recommended considering echocardiography in cases where a stroke mechanism has not been identified (Evidence Level C) or a cardiac cause of stroke is suspected, in patients with suspected embolic stroke and normal neurovascular imaging (Level B) and no contraindications for anticoagulant therapy [9].

About one out of five strokes is cardio-embolic in nature and stroke-recurrence rates are comparably high after cardio-embolic stroke. A cardiac source of embolism is more likely if multiple acute or subacute strokes in different vascular territories are detected, secondary hemorrhagic transformation is observed or a Valsalva maneuver preceded symptom onset [10]. There are several reasons why cardiac imaging should be performed in patients after ischemic stroke or TIA. First, most stroke patients are cardiovascular high-risk patients and the likelihood of alterations in cardiac structure and/or function is comparably high. Second, cardiac imaging helps to identify the most probable cause of ischemic stroke or TIA and subsequently lead to changes in secondary stroke prevention [11–13]. Third, imaging findings may guide further diagnostic management in stroke patients, e.g. intensified search for atrial fibrillation (AF) [14–16]. Fourth, certain imaging findings may have immediate therapeutic implications, e.g. endocarditis [17, 18].

This expert opinion paper collates and weighs the prevalence of stroke-related cardiac pathologies and most commonly used cardiac imaging methods with a focus on left atrial imaging. Summarizing present knowledge, expert-based suggestions for practical application of cardiac imaging after ischemic stroke or TIA are provided. Using the Delphi method, experts answered questionnaires in two rounds. Given (key point) recommendations are labeled as follows: ** if all experts agreed; * if the vast majority (≥ 90%) of experts agreed; without * if the majority (≥ 60–89%) of experts agreed.

## Prevalence of cardiac abnormalities in stroke patients

**Fig. 1:**
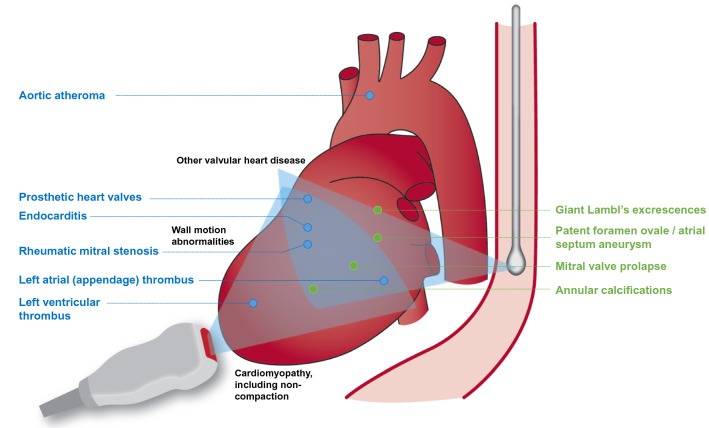
Scheme of the different topographies of the ultrasound probes and example imaging planes to illustrate differences in the ability to visualize cardiac structures, modified from [35, 36]. Common potential sources of embolism (high risk—blue, minor/unclear—green) or indicators of cardiovascular disease (black) are shown. Further, less common potential sources of embolism and echocardiographic findings are provided in Table 1

Cardiac and aortic sources of cerebral embolism resulting in stroke/TIA are heterogeneous [19]. A potential cardiac source of stroke can be identified in about 30% of unselected ischemic stroke patients [20]. Cardiac pathologies with embolic risk appear to have a similar frequency in older and younger stroke patients [21, 22]. A systematic review summarizing the results of very heterogeneous studies on the prevalence of cardiac findings showed that the most frequent findings in stroke patients are patent foramen ovale (PFO) and atrial septal aneurysm (ASA) [4]. While a PFO is prevalent in about one-quarter of all humans, PFO prevalence is higher in younger stroke patients with otherwise cryptogenic stroke (44–54% in case series) [4, 23–26]. An ASA is detected in 4–20% of all stroke patients and is accompanied by a PFO in about 60% [23, 26, 27]. Left atrial (LA) appendage (LAA) thrombus is a rather rare finding in stroke patients with an estimated prevalence of about 3% according to recent meta-analyses with even lower detection rate in stroke patients presenting in sinus rhythm [28–30]. Spontaneous echo contrast in the left atrium is observed in a broad range from 2 to 15.5% of stroke patients. Its etiological relevance is poorly understood, and its visualization depends on the echocardiographic equipment, sedation of the patients, and machine settings, which may help explain the large heterogeneity [31, 32]. Whereas mitral valve abnormalities were reported in about 10% of all stroke patients, rheumatic heart disease was less common in Europe [4]. A significantly reduced left ventricular ejection fraction was found in 2–16.7% of all stroke patients [4, 33, 34]. An empiric classification predominantly based on old echocardiographic studies divides cardiac conditions into high risk or low/unknown embolic risk (Fig. 1) [35, 36]. Less common potential sources of embolism include degenerative mitral valve stenosis, cardiomyopathies including myocarditis and storage disorders (e.g. amyloidosis, Fabry disease), atrial and ventricular communications, aortic abnormalities (e.g. related to Libman Sacks), and vasculitis (e.g. Kawasaki disease).

Treatment strategy changes by the initiation of anticoagulation have been reported in 3.4–8.0% [37, 38].

## Cardiac imaging after ischemic stroke

### Echocardiography

The most widely used imaging technology in post-stroke patients is echocardiography, which can be performed by TTE or TOE. TOE usually has a higher accuracy for the detection of potential cardio-embolic sources. Due to the anatomical neighborhood of heart and esophagus the TOE probe comes close to the heart permitting use of high-frequency ultrasound probes (4.2–7.4 MHz) enabling a high spatial and temporal image resolution [22, 39]. The combination of both bedside procedures permits a comprehensive evaluation of cardiac structure and function in real-time (Fig. 1).

TOE is a safe examination if rare contraindications are respected [40]. In many patients, TOE is well tolerated with pharyngeal and esophageal local anesthesia. A mild sedation is often used for a more convenient introduction of the TOE probe into the distal esophagus and the stomach. However, the patient should still be able to cooperate, e.g. perform Valsalva maneuver for PFO detection. Close hemodynamic monitoring is required if sedatives are used [39]. TOE is well-suited to examine valvular abnormalities (e.g. caused by endocarditis) and perpendicular imaging planes as well as multidimensional imaging help to assess valve function despite artifacts produced by mechanical heart valves that are frequent in TTE examinations. Furthermore, TOE is the gold standard technique for imaging of the morphology and structure of the atria including the interatrial septum, and the LAA, which is usually not visualized by TTE [22, 41]. Sometimes, the diagnosis of a PFO and atrial communication defects can be made by TTE if sufficient quality images can be generated.

TTE is non-invasive and well suited for the assessment of the morphology and function of cardiac cavities, especially the left ventricle and left atrium, as well as for the assessment of hemodynamically significant valvular pathologies. Contrast-enhanced TTE with ultrasound agent opacification of the cavities of the left heart can improve endocardial delineation, exclude left ventricular thrombi and support ischemia diagnosis during stress echocardiography. It should be performed in patients with poor acoustic windows or the suspicion of a left ventricular thrombus. TTE is limited by impaired ultrasound penetrance, especially in obese individuals or patients with pulmonary disease.

**Key points**

- Echocardiography is feasible and safe in patients with (acute) ischemic stroke or TIA.**
- TTE and TOE have to be considered as complimentary methods that provide distinct aspects of cardiac pathologies.**
- TOE is the method of choice for refined imaging of the atria and the interatrial septum. Details of (peri-)valvular abnormalities can be diagnosed with higher accuracy.**
- TTE is able to assess global and regional wall motion, ventricular abnormalities and left atrial function.**

**Fig. 2:**
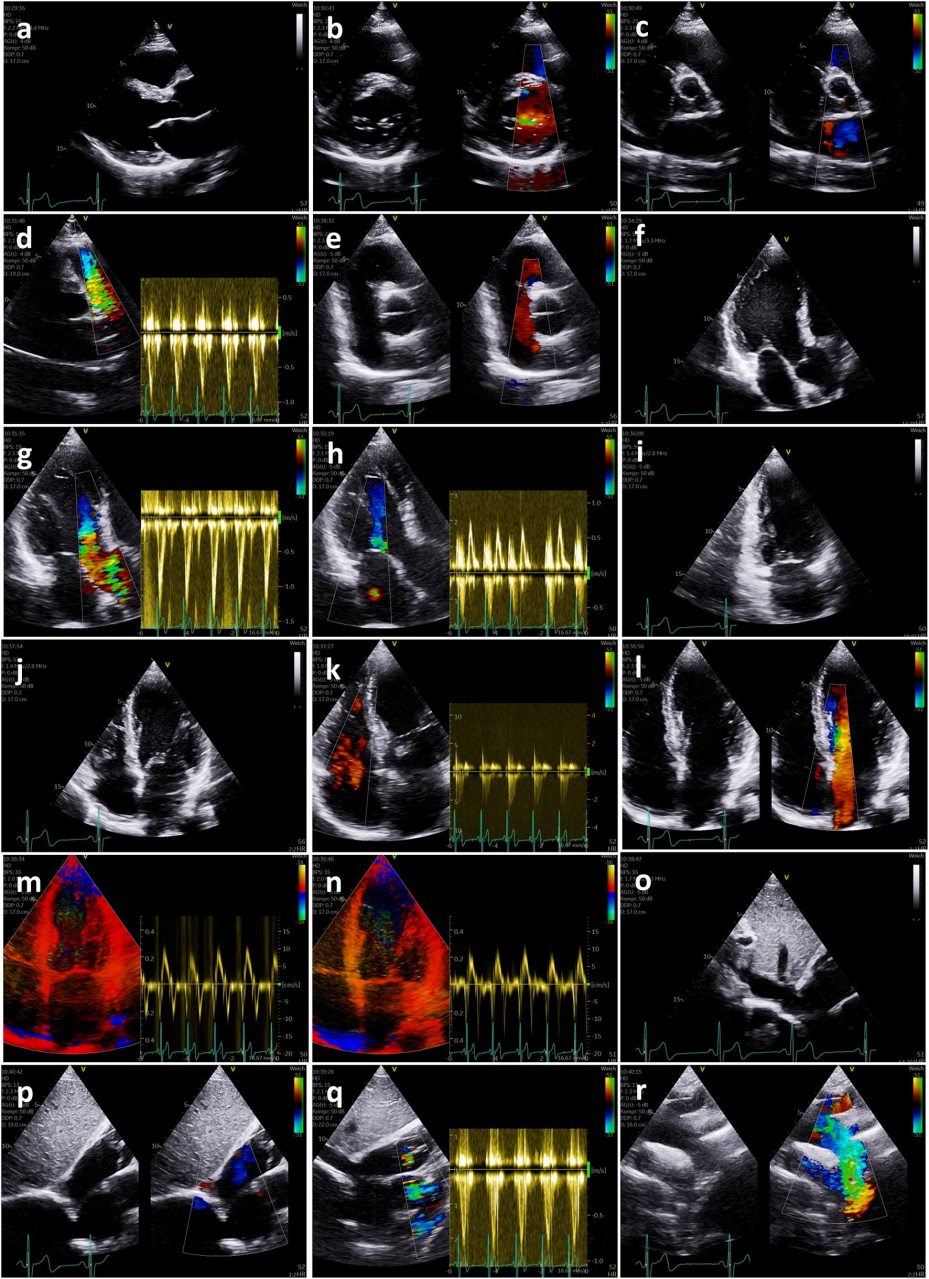
Image acquisition transthoracic echocardiography protocol in post-stroke patients. Parasternal long axis view for measurements of left ventricular (LV) diameters, left ventricular outflow tract (LVOT) diameter, LV-wall thickness, dimensions of the aortic arch (**a**); parasternal short axis view at the level of the mitral valve (MV) for assessment of MV pathologies, detection or exclusion of MV stenosis or MV regurgitation (**b**); parasternal short axis view at the level of the aortic valve (AV) for assessment of AV pathologies, detection or exclusion of AV stenosis or AV regurgitation (**c**); parasternal short axis view at the level of the pulmonary valve (PV) and pulsed wave (pw) Doppler spectrum of the right ventricular (RV) outflow tract (RVOT) flow to assess RV stroke volume for pulmonary stroke volume (Qp)/ systemic stroke volume (Qs)—calculation (**d**); parasternal short axis view of the interatrial septum to detect or exclude atrial communication defects (**e**); apical long axis view for assessment of LV function using deformation imaging (**f**); color-coded apical long axis view for assessment of AV function including pw Doppler spectrum of the LVOT flow to assess LV stroke volume for Qp/Qs-calculation (**g**), if AV is pathological a continuous wave (cw) Doppler spectrum has to be added; color-coded apical long axis view for assessment of MV function including pw Doppler spectrum of the transmitral flow (**h**), if MV is pathological a cw Doppler spectrum has to be added; apical 2-chamber view for assessment of LV function using deformation imaging (**i**); apical 4-chamber view for assessment of LV function using deformation imaging (**j**); color-coded apical 4-chamber view for assessment of tricuspid valve function including cw Doppler spectrum to assess systolic pulmonary artery pressure (sPAP) (**k**); color-coded apical 4-chamber view of the interatrial septum to detect or exclude atrial communication defects (**l**); color-coded tissue Doppler apical 4-chamber view including tissue pw Doppler spectrum of the basal septal myocardial velocities (**m**); color-coded tissue Doppler apical 4-chamber view including tissue pw Doppler spectrum of the lateral septal myocardial velocities (**n**); subcostal view of the inferior caval vein to document systemic volume state (**o**); subcostal short axis view of the interatrial septum to detect or exclude atrial communication defects (**p**); subcostal short axis view at the level of the pulmonary valve and pw Doppler spectrum of the RVOT flow to assess RV stroke volume for Qp/Qs-calculation, if parasternal view is not possible (**q**); suprasternal view of the aortic arch to detect or exclude aortic dissection and other aortic pathologies (**r**). *AV* aortic valve, *cw* continuous wave, *LV* left ventricular, *LVOT* left ventricular outflow tract, *MV* mitral valve, *pw* pulsed wave, *Qs* systemic stroke volume, *Qp* pulmonary stroke volume, *RV* right ventricular, *RVOT* right ventricular outflow tract

**Fig. 3:**
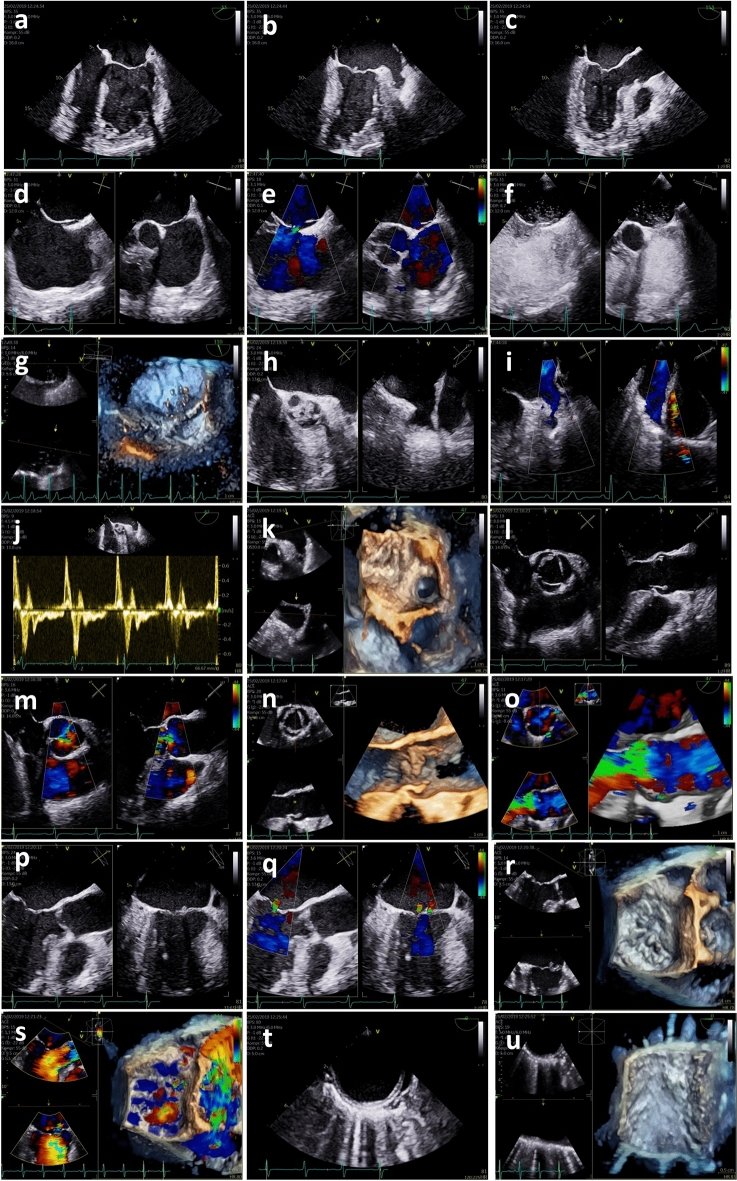
Image acquisition transoesophageal echocardiography protocol in post-stroke patients. Midoesophageal 4-chamber view for assessment of LV function using deformation imaging, if documentation using transthoracic echocardiography (TTE) is not possible (**a**); midoesophageal 2-chamber view for assessment of LV function using deformation imaging, if TTE documentation is not possible (**b**); midoesophageal long-axis view for assessment of LV function using deformation imaging, if TTE documentation is not possible (**c**); conventional 2D-documentation at least in 2 different sectional planes of the interatrial septum (IAS) (**d**); color-coded 2D-documentation at least in 2 different sectional planes of the IAS to document interatrial communication defects (**e**); contrast 2D-documentation with agitated saline at least in 2 different sectional planes of the IAS to document patent foramen ovale (PFO). Ideally no or mild sedation of the patient is performed and the Valsalva maneuver practiced with the patient before administration of agitated saline contrast agent. If available, 3D transoesophageal probes should be used and the test repeated several times, if negative. Good documentation is necessary to distinguish inter-atrial from trans-pulmonary shunts (**f**); contrast 3D-documentation with agitated saline to document PFO, if possible (**g**); conventional 2D-documentation at least in 2 different sectional planes of the left atrial appendage (LAA) (**h**); color-coded 2D-documentation at least in 2 different sectional planes of the LAA to exclude or document LAA thrombus formation (**i**); pulsed wave (pw) Doppler spectrum of the LAA flow velocities (**j**); 3D-documentation of the LAA, if possible (**k**); conventional 2D-documentation of the long axis and short axis view of the aortic valve (AV) (**l**); color-coded 2D-documentation of the long axis and short axis view of the AV (**m**); 3D-documentation of the AV and the aortic root complex, if possible (**n**); color-coded 3D-documentation of the AV and the aortic root complex, if relevant AS or AR is present and if possible (**o**); conventional 2D-documentation of the long axis and short axis view of the mitral valve (MV) (**p**); color-coded 2D-documentation of the long axis and short axis view of the MV (**q**); 3D-documentation of the MV, if possible (**r**); color-coded 3D-documentation of the MV, if relevant MS or MR is present and if possible (**s**); conventional 2D-documentation of the descending aorta/aortic arch (**t**); 3D-documentation of the descending aorta/aortic arch, if possible (**u**). *AR* aortic valve regurgitation, *AS* aortic valve stenosis, *AV* aortic valve, *IAS* interatrial septum, *LAA* left atrial appendage, *LV* left ventricular, *MR* mitral valve regurgitation, *MS* mitral valve stenosis, *MV* mitral valve, *PFO* patent foramen ovale, *pw* pulsed wave, *TTE* transthoracic echocardiography

Systematic imaging and documentation of a comprehensive, standardized echocardiography work-up is illustrated in Fig. 2 (TTE), Fig. 3 (TOE) and Supplementary Table 1.

With the rapid advancement of imaging methods with increasingly high resolution and short image acquisition duration, other modalities such as cardiac CT/CT angiography as well as cardiac MRI have become available for diagnostics after stroke. A semi-quantitative comparison of the different imaging modalities and specific indications is provided in Table 1. Cardiac CT carries the burden of a comparatively high radiation dose despite a number of dose reduction strategies that have been introduced over the last years [42–44]. Cardiac MRI comes at higher costs and requires comparatively long image acquisition times [45].

**Table 1:** Cardiovascular imaging modalities and their indications in stroke patients (modified from [3])

	TTE	TOE	CT	MRI
Availability	Widely available (bedside)	Widely available (fasting required)	Widely available	Limited capacities
Invasiveness	Non-invasive	Semi-invasive	Radiation exposure	Non-invasive
Resolution	Good spatial & excellent temporal resolution (potentially impaired by patient’s characteristics)	Excellent spatial & temporal resolution	Excellent spatial resolution & good temporal resolution^a^	Good spatial resolution & excellent tissue characterization
Costs	Comparatively cheap	Moderate	Comparatively expensive	Comparatively expensive
Operator dependence	High	High	Low	Low
Level of required patient cooperation	Comparatively low	Comparatively high (may require sedation)	Comparatively low	Comparatively high
Duration	Fast acquisition	Longer acquisition time	Fast acquisition	Longer acquisition time
Contrast agent	(Echocardiographic contrast agent)	(Echocardiographic contrast agent)	Iodinated contrast agent	Gadolinium exposure
Structures/Pathologies				
Left ventricular				
Dysfunction	+ + +	+ +	+	+ + +
Thrombus	+ +	+	+ +	+ + +
Cardiomyopathies	+ + +	+	+	+ + +
Coronary artery disease ^b^	+ +	+	+ + +	+ +
Left atrial				
Morphology	+ +	+ + +	+ + +	+ +
Dysfunction	+ + +	+ +	+	+ +
Thrombus	+ +	+ + +	+ + +	+ +
Left atrial appendix				
Morphology	+	+ + +	+ + +	+
Dysfunction	+	+ + +	+	+
Thrombus	+	+ + +	+ + +	+ +
Interatrial septal defects	+ +	+ + +	+	+
Intracardiac shunt	+ +	+ +	+	+ +
Endocarditis	+ +	+ + +	+ + + ^c^	+
(Peri-)valvular disease	+ +	+ + +	+ + +	+ +
Valvular calcifications	+ +	+ +	+ + +	+ +
Cardiac tumors	+ +	+ +	+ + +	+ + +
Thoracic aorta				
Morphology	+	+ +	+ + +	+ +
Plaques	+	+ +	+ + +	+ +
Dissection	+	+ + +	+ + +	+ +

*CT* computed tomography, *MRI* magnetic resonance imaging, *TOE* transoesophageal echocardiography, *TTE* transthoracic echocardiography

+  +  + Very good, +  + good, + moderate/weak

^a^Depending on CT scanner and acquisition mode

^b^Cardiac CT is the only non-invasive imaging technique, which is clinically established for the anatomical assessment of coronary artery disease

^c^In combination with positron emission tomography

### Cardiac computed tomography

ECG-gated cardiac scanning with multi-detector 64-slice CT systems that scan large volumes at high speed (short breath hold) the temporal and spatial resolution significantly improves and enables visualization of small and volatile structures [46]. The use of ECG-gated imaging protocols has led to a significant decrease in the radiation dose for cardiac CT with a pooled radiation dose of 3.5 mSv according to a meta-analysis from 2013 [43, 44] and German cardiac CT registry experience [47]. Contrast-enhanced cardiac CT can produce high-quality images of the cardiac walls and lumen, the coronary arteries and the large vessels including the aortic arch and descending aorta. In a meta-analysis, LA and LAA thrombi were detected with a sensitivity of 96% and a negative predictive value of 99% compared to the gold standard TOE, which permits rule-out [48]. Its specificity for thrombi is limited by pseudo-filling defects due to blood stasis, which can be optimized by using two-phase or dual-enhanced computed tomography image acquisition, i.e. including late-phase images [49]. Thus, in a meta-analysis from 2013 cardiac CT exhibited reliable diagnostic characteristics for the detection of atrial clots, when delayed imaging is performed [48]. In addition, dual-energy cardiac CT acquiring images at different energy levels may improve the detection of LAA thrombus by differentiating clots from slow blood flow, obviating the need for delayed imaging and thus additional radiation exposure [50].

Furthermore, cardiac CT can contribute to the differentiation of left ventricular thrombi and other intraventricular masses due to its higher spatial resolution and improved delineation of endocardial borders, but CT usually follows initial evidence from an echocardiogram in the clinical routine [51, 52]. The latter remains the standard for follow-up examinations. CT has the advantage of virtually unrestricted imaging views with isotropic spatial resolution [51, 52]. On the other hand, cardiac CT generates limited information on tissue characteristics compared to MRI and relies on anatomical features to suggest the tumor entity. The diagnostic accuracy for PFO and ASA is limited because small, mobile structures are not well delineated and PFO diagnosis usually requires Valsalva maneuver [53]. In patients with suspected or proven endocarditis, CT imaging can reveal life-threatening perivalvular complications such as abscess or mycotic aneurysms, which is of particular importance in patients with prosthetic heart valves, increasingly transcatheter heart valve replacement [54]. Functional imaging by positron emission tomography (PET) combined with CT uses higher metabolic activity in inflammatory tissue for the diagnosis of endocarditis [55]. So far, no direct comparisons for TOE versus CT or PET-CT for the diagnosis of endocarditis and its complications are available. CT may also support the diagnosis and quantification of the extent of valvular calcifications as potential sources of embolism [56]. In addition, CT angiography or calcium scoring are clinically established for the diagnostic classification and risk stratification of patients with suspected or known coronary artery disease (CAD) [57, 58] However, little data exist on its diagnostic or prognostic value in post-stroke patients [59].In addition, modern methods including CT-derived functional flow reserve and CT perfusion may help to also evaluate ischemia [60]. In stroke patients, cardiac CT is a feasible alternative, but diagnostic accuracy compared to TOE is limited as outlined above [53, 61–64].

**Key points**

- Cardiac CT is feasible in stroke patients due to the short acquisition time; however, it comes at the cost of radiation exposure.*
- Cardiac CT can be a complementary imaging method for additional work-up of specific patients with acute ischemic stroke or TIA, but is not optimal for the evaluation of most frequent cardiac sources of embolism.*

### Cardiac MRI

Cardiac MRI is emerging as a non-invasive tomographic imaging method for post-stroke cardiac work-up [45, 65]. Today, cardiac MRI requires comparatively long image acquisition times and affords the patient to follow breathing commands, limiting the feasibility in stroke patients. This may be overcome by modern non-breath-hold and compressed sequences available for CINE and late gadolinium enhancement (LGE) imaging. MRI has become the gold standard for ventricular volumes and mass. It can visualize complex cardiac abnormalities and quantify hemodynamics including valve disease and permits excellent soft tissue characterization. Stress MRI can assess cardiac ischemia and LGE uses cellular integrity to show myocardial viability, fibrosis, and scarring. It may be superior to echocardiography in the diagnosis of previous (clinically silent) myocardial infarction. Delayed-enhancement cardiac MRI is the gold standard for left ventricular thrombus detection due to its excellent tissue delineation with an absence of contrast enrichment in thrombus material [66, 67].

After initial TTE, cardiac MRI can further aid to differentiate unclear, less common cardiomyopathies, e.g. in case of cardiac amyloidosis or non-compaction cardiomyopathy [68–70]. In a recent two-center study, cardiac MRI detected seven cases of prior unknown cardiomyopathy in 132 patients with ischemic stroke and no cardiac source of embolism on TTE [71]. While the detection rate of LA thrombi correlates well with TOE, the diagnosis of LAA structures, evaluation of the interatrial septum and PFO has remained technically challenging and is not reliable yet [45, 72].

The feasibility of cardiac MRI in consecutive stroke patients has been demonstrated in a single-center study as 89 of 103 patients with acute ischemic stroke completed the 50-min examination [45]. The use of cardiac MRI reduced the number of strokes that remained cryptogenic at the end of in-hospital diagnostics through the identification of additional potential cardioembolic sources, mainly regional wall motion abnormalities in more than three segments. Furthermore, previously undetected non-acute myocardial infarction was detected in 15% of all cryptogenic stroke patients [45]. Application of cardiac stress MRI is not established in the acute phase of stroke.

**Key points**

- Cardiac MRI is a complementary imaging method for work-up of specific cardiac pathologies, for example tumors, cardiomyopathies and left ventricular thrombi or prior myocardial infarction.**
- Cardiac MRI is not optimal for the evaluation of PFO or the detection of endocarditis.
- In general, cardiac MRI requires comparatively long image acquisition times and affords the patient to follow commands, limiting the feasibility in acute stroke patients.*

## Left atrial imaging in stroke patients

The importance of left atrial imaging increases as the concept and clinical relevance of atrial (cardio-) myopathy evolves. Atrial cardiomyopathy is defined by the presence of structural, architectural, contractile or electrophysiological changes and may carry an increased risk of embolic stroke independent of (ECG detected) AF [73, 74]. Anatomical and functional parameters of the left atrium add prognostic information beyond established risk markers of increased mortality in the older community [75]. Left atrial dilatation is associated with increased stroke risk [76]. In addition, LAA size and morphology are also associated with increased stroke risk [77]. Thus, the characterization of LAA morphology by 3D imaging techniques to detect potential thrombi in the LAA lobi is important in post stroke patients. One problem in this patient cohort is, that a thrombus formation may not be detectable anymore at the moment of the imaging procedure, as the thrombus has led to stroke or residual thrombus formation dissolved spontaneously or by antithrombotic treatment. The target of TOE imaging in this scenario is the analysis of the functional state of the LAA, mainly characterized by LAA emptying velocities. Left atrial shape is another variable to assess thrombogenicity [78, 79]. The application of contrast agents can help differentiate between spontaneous echo contrast and solid thrombi. Tissue Doppler derived markers and strain imaging are promising methods to assess atrial performance and help quantify atrial cardiomyopathy. New echocardiographic techniques include left atrial reservoir strain, which is easy to perform and adds prognostic information, as reported in patients with AF [80]. Decreased left atrial strain is thought to reflect left atrial fibrosis [81–83]. LA function can also be measured using cardiac MRI and CT [84–87]. Ongoing studies like ATTICUS and ARCADIA focus on the potential role of left atrial pathology for recurrent stroke risk after ESUS and investigate, whether secondary stroke prevention using apixaban is superior/non-inferior to acetylsalicylic acid [88, 89]. As (also clinically unapparent) AF leads to atrial fibrosis, left atrial imaging may be applied to tailor intensity of screening for intermittent AF [90].

**Key points**

- Left atrial imaging may be used to assess atrial cardiomyopathy.*
- Presence of atrial cardiomyopathy can help to tailor the intensity of post-stroke monitoring for AF.**

## Practical recommendations to use cardiac imaging after ischemic stroke or TIA

Careful work-up is necessary to identify potential cardiac sources of embolism. Furthermore, stroke work-up provides an opportunity to screen for cardiac comorbidities that may prompt further diagnostic evaluation and affect treatment that improves cardiovascular outcome.

In Germany, there is broad consensus to perform echocardiography in selected patients with acute ischemic stroke, as registry data demonstrated an increased use of echocardiography from 62.2% in 2001 to 74.0% in 2006 [91]. This is in line with registry data from Canada showing that the proportion of stroke patients undergoing echocardiography rose from 52% in 2003/2004 to 70% in 2011/2012 [92]. The German Stroke Society (DSG) certification criteria for German Stroke Units require the use of TOE in at least 15% of all ischemic stroke patients (aiming for 20–30%), considering TOE superior to TTE with regard to diagnostic power [93]. The German Cardiac Society (DGK) as well as international societies recommend cardiac MRI or CT in addition to TTE/TOE rather than method of first choice if there is a suspicion for a specific condition/pathology amenable for MRI or CT imaging [19, 60, 94, 95].

An optimal secondary stroke prevention strategy may also affect health economics. However, no prospective randomized study has proven that secondary prevention measurements (e.g. oral anticoagulation) are efficacious with regard to clinically relevant endpoints. Furthermore, there is only limited data available on how often echocardiography leads to relevant changes in therapeutic management. Observational studies, mainly focusing on changes in secondary stroke prevention, suggest that this is only rarely the case, rendering a reduction of stroke recurrence or reduction of other (vascular) endpoints unlikely [37, 38, 96, 97]. However, given the recently published open-label randomized trials demonstrating a benefit of interventional PFO-closure over antiplatelet agents in cryptogenic stroke patients aged 16–60 years [98–100], the impact of echocardiography is likely to increase.

### Setting for echocardiography

An interdisciplinary team routinely collaborating on the cardiology work-up of post-stroke patients may facilitate cross-discipline communication and improve patient care. Ideally, a specialized cardiovascular imaging suite is nearby or even integrated in the stroke unit, which permits short transportation times or bedside echocardiography. If post stroke echocardiography is performed in a cardiology routine environment, fixed slots for stroke unit patients may facilitate logistics. Echocardiographic work-up should ideally be finalized during the hospital stay to ensure an interdisciplinary approach for patient discussion and potential further diagnostic or therapeutic decisions. Therefore, adequate structures and resources are necessary for stroke-care hospitals. In case of limited echocardiographic capacities and short duration of in-hospital stays, echocardiographic work-up can be performed post-discharge in patients in stable cardiac conditions with an established non-cardiac cause of stroke.

The quality of echocardiographic findings is investigator-dependent [101, 102]. This variability is composed of differences in image acquisition and image analysis [101]. In particular, there seems to be a significant intra- and interobserver variability in the diagnosis and quantification of PFO, spontaneous echo contrast and left atrial thrombi with TOE [103–105]. Therefore, TTE and TOE should be performed by an experienced cardiologist, ideally supported by a nurse. A senior cardiology consultant should be available to advise the stroke unit team on specific work-up based on imaging findings.

**Key points**

- A standardized set-up and experienced investigators are needed for echocardiographic examinations in stroke patients, which should follow a systematic, standardized protocol.*
- An interdisciplinary team and *standard operating procedures* for post-stroke cardiac imaging may enhance decision making and advise further work-up including special cases where cardiac CT or MRI are required.*

### Patient selection

Cardiac imaging should be performed if it has potential therapeutic consequences. In case of a defined non-cardiac source of stroke (e.g. arterial dissection) and a low cardiovascular risk profile, cardiac imaging is not essential after stroke or TIA.

**Fig. 4:**
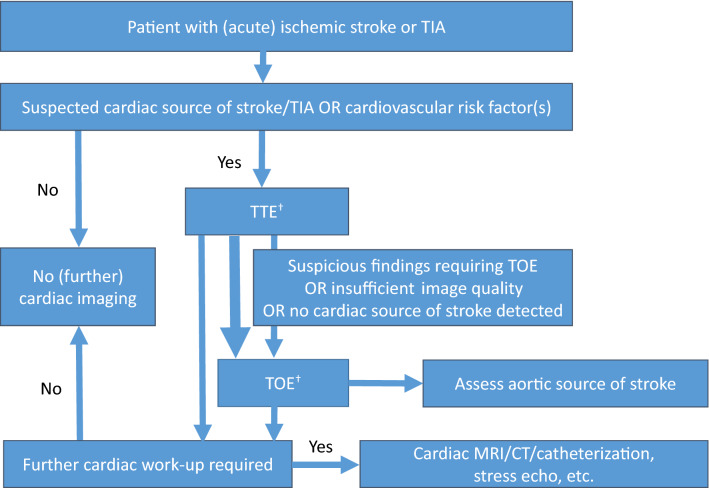
Expert-based recommendations for post-stroke imaging. Patients with (acute) ischemic stroke or TIA should receive transthoracic echocardiography (TTE) if either a cardiac cause of stroke is suspected or any cardiovascular risk factor is present. Transoesophageal echocardiography (TOE) should additionally be performed in patients with suspicious findings, insufficient image quality or if no cardiac source of stroke was detected despite clinical suspicion for cardioembolic stroke (†TTE and TOE in a single session is recommended if the cardiac source of stroke is suspected as indicated by the bold arrow). Inconclusive TTE or TOE and specific suspicion of cardiac disease may lead to further diagnostic work-up, e.g., contrast-enhanced echocardiography or cardiac magnetic resonance imaging. The value of different cardiovascular imaging methods for common cardiac pathologies is listed in Table 1

Since guidelines recommendation consistently encourage echocardiography in patients with suspected embolic stroke and without contraindications for oral anticoagulation, this approach selects in particular younger patients with stroke or TIA. As cardiac comorbidities such as coronary heart disease, left ventricular hypertrophy, and valvular dysfunction naturally becomes more common in older age groups, expert consensus is that TTE should be considered in patients with at least one established cardiovascular risk factor (Fig. 4).

Acute ischemic stroke or TIA is an indicator of a comparably high risk of cardiovascular co-morbidities that may not be directly causally related to the present stroke but may significantly affect stroke recurrence rate and survival [106]. In particular, in older patients with acute ischemic stroke, a high cardiovascular risk factor burden is evident [107, 108]. In addition, coronary heart disease is highly prevalent in patients with stroke/TIA or carotid atherosclerosis and should actively be risk-stratified [109–112]. Cardiac imaging should therefore not be restricted to (comparably young) patients with so far cryptogenic stroke or ESUS [113]. Whereas echocardiographic work-up is generally recommended in TIA patients [114], it is expert consensus that in low-risk TIA patients (i.e. without a history of cardiac disease or stroke, normal cardiac physical examination and normal ECG during monitoring), imaging may seldom result in relevant findings [115]. If a recent cardiologic work-up is documented and clinical status is unaltered, cardiac imaging can be postponed post-hospital discharge, if cardio-embolic source is known already or not expected. Patients at high risk for intracardiac thrombi are individuals with AF which is observed in more than 20% of acute ischemic stroke patients from hospital registries with current rhythm monitoring strategies [85]. However, additional relevant sources of embolism, like a ventricular thrombus or severe atherosclerotic plaques in the aortic arc can be found in AF patients [11, 12]. Furthermore, AF often is associated with additional cardiac abnormalities such as heart failure or valvular heart disease, which are not detected on physical examination, but merit diagnostic attention and therapeutic optimization [11, 17, 18]. TTE should be performed in patients if the presence of heart failure or changes of cardiac function in patients with heart failure would alter further management. The presence of a ventricular thrombus indicates a separate cardiac source of embolism and an LA(A) thrombus may lead to early continuation or starting of oral anticoagulation [11, 12].

**Key points**

- Stroke or TIA patients should undergo cardiac imaging, if stroke etiology is uncertain.*
- Stroke or TIA patients should undergo cardiac imaging, if the presence of pathological findings would alter (medical) management.*
- Cardiac imaging should be considered in ischemic stroke patients with at least one established cardiovascular risk factor to identify cardiac comorbidities, unless cardiologic work-up within the last 6 months is documented in stroke patients without signs of cardiac dysfunction.
- Stroke or TIA patients with AF should undergo cardiac imaging for further cardiac work-up, if the presence of a left-sided atrial or ventricular thrombus would alter (medical) management or if a first episode of AF was documented in-hospital.*

### Imaging method of choice

Whereas routine echocardiography in all stroke patients is not recommended, guidelines consistently encourage echocardiography in patients with suspected embolic stroke with unsuspicious neurovascular imaging and no contraindications against anticoagulation where a diagnosis would lead to a treatment change [1, 6, 8, 9].

For the diagnosis of atrial sources of emboli TOE is necessary. However, in the vast majority of cases, patients with cardiac thrombi have underlying functional or structural cardiac alterations (e.g. reduced left ventricular ejection fraction for ischemic heart disease or cardiomyopathy, mitral valve stenosis or previously documented AF). If any of these conditions is absent, the 12-lead ECG is normal, and no history of cardiovascular disease is present left atrial thrombi are a rare finding [30, 31, 116]. The diagnosis of PFO has gained more importance, as three recent RCTs demonstrated a benefit of PFO closure over antiplatelet therapy in stroke patients aged 16–60 years with at least moderate shunt [98–100]. TOE with an intravenously injection of an agitated air-saline or air-modified gelatin solution (“bubble-test”) is the gold standard of PFO detection and semi-quantification [117]. The extent of intermittent right-left-shunting through the PFO sometimes is difficult to quantify by left atrial bubble transfer, because the opening of the PFO is dependent on the pressure conditions between the right and left atrium. Thus, in the presence of a severe increased left atrial pressure (e.g. severe diastolic dysfunction or severe aortic stenosis) a PFO may not be detectable by bubble passage, even if it is large. Initial screening for a right-to-left shunt can be achieved using contrast transcranial Doppler examination to monitor potential microbubbles in the middle cerebral arteries [118].

Increased left atrial dimensions or parameters of reduced left atrial function summarized as atrial cardiomyopathy may hint towards paroxysmal AF as a potential cause of stroke, reduced left ventricular ejection fraction, and regional wall motion abnormalities towards left ventricular thrombus [14, 73, 119]. Stroke patients with the first episode of AF in-hospital should undergo TTE, as preexisting heart disease underlies newly detected AF according to a cohort study [79, 120].

In stroke patients with known AF prior to stroke, a TTE should be considered (unless cardiac work-up is documented within the last 6 months and cardiac clinical state has not changed). Furthermore, a TOE should be performed in stroke patients with AF, if the presence of an atrial thrombus would alter medical management, e.g. lead to immediate oral anticoagulation despite the risk of secondary hemorrhagic transformation.

Cardiac CT imaging is suitable for the diagnostic classification of patients with suspected coronary artery disease and cardiovascular risk stratification [58–60]. However, there is no broad evidence (or consensus), which stroke patients (without suspected endocarditis) may benefit from additional imaging using cardiac CT. Regarding the additional radiation dose, cardiac CT should only be considered as an imaging modality for individual cases in which relevant information cannot be obtained by echocardiography or cardiac MRI [121]. Despite high sensitivity for thrombus detection, long image acquisition times, limited availability (even in centers with specific expertise), needed ability of the patient to follow breathing commands and comparably high costs of cardiac MRI limit its applicability outside the setting of prospective studies. Furthermore, impaired renal function frequently observed in post-stroke patients limits the application of gadolinium-contrast agents or CT-contrast agents [122].

For now, it appears that cardiac CT and MRI may serve as complementary imaging modalities in clinical studies or for in-depth work-up in case of suspected cardiac abnormalities that remain inconclusive in echocardiographic examinations. CT/MRI can also be applied in rare cases where contraindications against TOE are present or image interpretability is impaired. The use of either method will not only depend on the local expertise but largely on the availability of high-quality imaging facilities. One may argue that chest x-ray is routinely accessible in the stroke unit setting, and can reveal cardiac enlargement, pulmonary congestion, pleural effusion and even valve calcifications. As no respective studies are available [123], it is expert consensus that chest x-ray is not sufficient to substitute for echocardiography, cardiac MRI or CT imaging.

**Key points**

- Echocardiography is the gold standard imaging modality after ischemic stroke or TIA, if a pathological finding would alter (medical) management.*
- TOE and TTE should be performed (preferably in a single session) if a cardio-embolic source is deemed a probable cause of ischemic stroke or TIA.
- TTE should be performed in patients with suspicious findings during TOE, if TOE alone is not sufficient for the comprehensive evaluation of the specific pathology (e.g. regional wall motion abnormalities or apical left ventricular thrombus).
- TOE should be performed in patients with suspicious findings during TTE, if TTE alone is not sufficient for the comprehensive evaluation of the specific pathology (e.g. endocarditis or assessment of (inter)atrial structures).**
- TOE should be performed (in patients with so far cryptogenic stroke aged 16–60 years), if PFO presence would alter further management.*
- TTE should be performed in stroke patients with a first episode of AF in-hospital.*
- TTE should be considered in ischemic stroke patients with at least one established cardiovascular risk factor unless recent cardiologic work-up is documented.*
- Cardiac CT should be considered, if there is a suspicion of cardiac or extra cardiac abnormalities, based on other imaging modalities, which are assumed to be clinically relevant. As cardiac CT is associated with radiation exposure, use of cardiac CT should be based on an individual decision.*
- Cardiac MRI should be considered, if there is a suspicion of left ventricular thrombus or cardiac tumor or in case of an unclear cardiomyopathy after contrast enhanced echocardiography.

## Incidental imaging findings

With intensified cardiac imaging, work-up of incidental findings needs to be considered. Such findings may result in uncertainty on diagnostic and treatment consequences in often older and multi-morbid patients. In stroke patients a PFO is a frequent (most often incidental) finding due to the high prevalence in the general population [24]. Frequent incidental findings also include aortic atheroma/plaques, calcification of the aortic/mitral valve or spontaneous echo contrast. Therapeutic decisions should be evidence-based and ideally follow standard operation procedures. An interdisciplinary board may help in efficacious and swift interdisciplinary decisions.

**Key points**

- Diagnostic algorithms and interdisciplinary standard operating procedures should be established to avoid over-diagnosis and “over-treatment” because of incidental findings of cardiac imaging.*

## Cost-effectiveness of cardiac imaging after stroke

Overall, cost-effectiveness analyses are impaired by the limited data on the effectiveness of treatment for most imaging findings. At present, reliable numbers exist for left atrial appendage thrombus and subsequent OAC only and will probably soon be available for PFO closure [124]. Whereas cost-effectiveness may not be given if performed in unselected acute ischemic stroke patients, modern TTE has been classified as cost-effective compared to no testing when used for suitable indications in post-stroke patients across age groups between 45 and 65 years [1, 4]. These cost calculations neglected a potential benefit from incidental findings related to other common cardiovascular diseases that may be treatable and thus add quality-adjusted life years. No cost–benefit calculations have been performed to assess the overall yield of echocardiographic imaging and treatment changes beyond the initiation of anticoagulation.

For other imaging methods such as cardiac CT or MRI implementation in acute stroke imaging protocols has been suggested, but robust data on the benefit beyond echocardiography are not available. Cardiac CT and in particular MRI examinations are significantly more expensive than echocardiography, with TTE being the cheapest of all cardiac imaging methods [125]. For all imaging modalities, most studies were comparatively small, single-center observational examinations. No systematic head-to-head comparisons including treatment change and outcomes are available. To date, cardiac CT or MRI have not been endorsed as the appropriate utilization of cardiovascular imaging in the setting of stroke [94, 121].

**Key points**

- Cost-effectiveness of systematic cardiac imaging in stroke patients should be addressed in prospective trials.

## Imaging of the aortic arch

Aortic arch atheromas have been related to recurrent embolic stroke [126]. A CT scan can visualize the whole aortic arch and the descending aorta whereas TOE often is impaired by suboptimal acoustic properties of the aortic arch [64]. Furthermore, novel CT imaging tools permit optimized aortic plaque characterization with delineation of its different components, ulcerations, adherent thrombi beyond plaque thickness and extension and therefore, render CT angiography superior to TOE for aortic plaque assessment [127, 128]. Multi-detector row CT detected atheroma may constitute a risk factor for stroke recurrence, although treatment strategies are not clear yet [127, 129]. MRI angiography can depict aortic atheroma features with limitations for calcifications, mobile structures and ulcerations [130, 131].

**Key points**

- A CT angiography scan can visualize the whole aortic arch and the descending aorta whereas TOE often is impaired by suboptimal acoustic properties of the aortic arch.
- MRI angiography can depict aortic pathology but acquisition is time-consuming, more expensive, and limited with regard to calcifications, mobile structures and ulcerations.*

## Summary

In conclusion, stringent high-quality cardiac imaging in ischemic stroke or TIA patients offers the opportunity to reveal stroke causes with direct therapeutic consequences and to improve global cardiovascular risk assessment in a high-risk population for cardiovascular disease. According to the working group, TTE should be considered in stroke patients with at least one established cardiovascular risk factor. A TOE should be performed, if a cardiac source of embolism is suspected or TTE findings need further work-up. Non-invasive cardiac imaging using CT or MRI can be viewed as complementary methods, but should currently be restricted to specific diagnostic questions and clinical studies.

## Supplementary Information

Below is the link to the electronic supplementary material.

Supplementary file1 (DOCX 75 kb)

## Abbreviations

ASA: Atrial septal aneurysm
CT: Computed tomography
ECG: Electrocardiogram
ESO: European Stroke Organization
ESUS: Embolic stroke of undetermined source
LA: Left atrium
LAA: Left atrial appendage
LGE: Late gadolinium enhancement
MRI: Magnetic resonance imaging
PFO: Patent foramen ovale
RCT: Randomized controlled trial
TIA: Transient ischemic attack
TOE: Transoesophageal echocardiography
TTE: Transthoracic echocardiography
