# Supplementary material for: Expert opinion paper on cardiac imaging after ischemic stroke
Source: Clin Res Cardiol. 2021 Jun 18;110(7):938–58. doi: 10.1007/s00392-021-01834-x (PMC8238761; doi:10.1007/s00392-021-01834-x)
Supplement: Supplementary file 1 — Supplementary file1 (DOCX 75 kb) [file 392_2021_1834_MOESM1_ESM.docx]

Expert opinion paper of the “Heart and Brain” consortium of the German Cardiac Society and the German Stroke Society

**Expert opinion paper on cardiac imaging after ischemic stroke**

Supplementary information

**Supplementary Table 1** Echocardiographic measurements in stroke patients.

| **Target** | **Parameter** | **Image sequence** | **Limitations** |
| --- | --- | --- | --- |
| Left ventricular (LV) hypertrophy | - Septal wall thickness - Thickness of the posterior wall - Relative wall thickness | - Parasternal long axis - If parasternal not possible, use apical long axis | - Bad parasternal acoustic window; - Non standardized sectional planes |
| LV dilatation | - LV endsystolic and enddiastolic diameters | - Parasternal long axis - If parasternal not possible, use apical long axis | - Bad parasternal acoustic window; - Non standardized sectional planes |
| Cardiac performance – effective stroke volume (LVSV_eff_ and RVSV_eff_);  Assessment of potential shunt volume Qp/Qs (systemic stroke volume/pulmonary stroke volume) | - Diameter of the LV outflow tract (LVOT) and velocity time integral of LVOT (VTI_LVOT_) - Diameter of the right ventricular outflow tract (RVOT) and velocity time integral of RVOT (VTI_RVOT_) | - Parasternal (or apical) long axis (LVOT) and pw spectrum of the LVOT flow - Parasternal (or subcostal) long axis view of the RVOT and pw spectrum of the LVOT flow | - Bad parasternal acoustic window; - Non standardized sectional planes - Incorrect position of the pw Doppler sample volume - Not representative in the presence of severe aortic valve (AV) regurgitation / pulmonary valve (PV) regurgitation - RVSV_eff_ error prone in patients with severe tricuspid valve regurgitation |
| LV performance – LV volumes (LVEDV, LVESV, total stroke volume and (LVSV_tot_), left ventricular ejection fraction | - LV areas at enddiastole and endsystole by LV planimetry (2-chamber and 4-chamber view) | - Apical 2-chamber view - Apical 4-chamber view - If transthoracic echocardiography (TTE) is not sufficient, transoesophageal echocardiography (TOE) documentation | - Non standardized, foreshortening sectional planes |
| Diastolic dysfunction:  E-velocity (maximum velocity at early diastolic inflow), E/A-ratio ( ratio between maximum velocity of early and late diastolic inflow), E`- velocity (maximum velocity of basal myocardial velocities – mean value of septal and lateral LV regions), E/E`- ratio ( ratio between maximum blood velocity of early diastolic inflow and corresponding myocardial velocities),  systolic pulmonary artery pressure) (sPAP),  size of the inferior caval vein and collapse index | - Transmitral pw-Doppler spectrum (E-velocity, E/A-ratio) - Tissue velocity pulsed-wave Doppler spectrum (E`- velocity, E/E`-ratio) - Transtricuspid cw Doppler spectrum (sPAP) - Diameter of the inferior caval vein and collapse index | - Color-coded apical long axis view and consecutive pulsed-wave-Doppler spectrum for transmitral flow documentation - Color-coded apical tissue Doppler 4-chamber view and consecutive pw-tissue Doppler spectrum for documentation of myocardial velocities - Color-coded apical 4-chamber view and consecutive continuous wave-tissue Doppler spectrum for documentation of transtricuspid regurgitant velocities - Subcostal long axis of the inferior caval vein | - Non standardized sectional planes - Incorrect position of the pw Doppler sample volumes - Not representative in patients with relevant mitral valve (MV) stenosis and/or septal and lateral wall motion abnormalities |
| Aortic valve (AV) | Qualitative description   - Cuspidity - Calcification - Vegetations - Thrombus formation - Tumors - Lambl`s excrescences | - Parasternal and apical long axis views - Parasternal and subcostal short axis views - TOE | - Bad acoustic window - Secants of the long axis view - If TTE is not clarifying the AV status, TOE is always necessary - Shadowing artifacts in AV prosthesis |
| Aortic root complex and aortic arch | Qualitative description   - Abscess formation - Dissection - Ectasia/Aneurysma - Plaques   Diameter of the aortic annulus (D_AV_), aortic root – Sinus of Valsalvae (D_SV_), sinotubular junction (D_STJ_), and proximal tubular ascending aorta (D_TAA_), as well as aortic arch (D_AA_) | - Parasternal long axis view during systole - Suprasternal view - TOE, if TTE is not sufficient to visualize the aortic root - TOE for documentation of the descending aorta | - Bad acoustic window |
| Mitral valve (MV) | Qualitative description   - Morphology of the leaflets - Pathologies of the mitral valve apparatus - Vegetations - Tumors - Abscess formation | - Parasternal long and short axis views - Apical long axis view and 2- and 4-chamber view short axis views - TOE documentation | - Bad acoustic window - If TTE is not clarifying the MV status, TOE is always necessary - Shadowing artifacts in MV prosthesis |
| Interatrial septum (IAS) | Qualitative description   - Patent foramen ovale - Thrombus formations, especially transit thrombi - Atrial septal aneurysm - Atrial septal defect - Tumors | - Color-coded TTE documentation using the parasternal, apical (especially, if ventricular septal defect is suspected) and/or subcostal view - TOE documentation at least in two sectional planes, preferably perpendicular - Bubble transfer after contrast administration should be documented | - Bad acoustic window - If TTE is not clarifying the IAS status, TOE is always necessary |
| Left atrium (LA) and left atrial appendage (LAA) | Qualitative description   - Thrombus formations, especially in the LAA - Spontaneous echo contrast and sludge formation in the LAA - LAA velocities | - Normally TOE documentation at least in two sectional planes, preferably perpendicular - pw Doppler spectrum of LAA velocities | - 3D documentation is preferred if excentric lobi or cactus LAA morphology is present - Low velocity reject and scale has to be adapted to maximum LAA velocities - If conventional TOE is not clarifying the LAA status, contrast for LAA opacification should be used. |
| AV, aortic valve; IAS, interatrial septum; LA, left atrium; LAA, left atrial appendage; LV, left ventricular; LVOT, left ventricular outflow tract; MV, mitral valve; PV, pulmonary valve; sPAP, systolic pulmonary artery pressure | | | |
